# Trends in the prevalence and management of diagnosed type 2 diabetes 1994–2001 in England and Wales

**DOI:** 10.1186/1471-2296-6-13

**Published:** 2005-03-22

**Authors:** Simon de Lusignan, Charalambos Sismanidis, Iain M Carey, Stephen DeWilde, Nicky Richards, Derek G Cook

**Affiliations:** 1 Department of Community Health Sciences, St George's Hospital Medical School, London SW17 0RE, UK; 2 CompuFile Ltd., 1 Tannery House, Tannery Lane, Send, Surrey GU23 7EF, UK

## Abstract

**Background:**

Type 2 diabetes is an important cause of morbidity and mortality. Its prevalence appears to be increasing. Guidelines exist regarding its management. Recommendations regarding drug therapy have changed. Little is known about the influence of these guidelines and changed recommendations on the actual management of patients with type 2 diabetes. This study aims to document trends in the prevalence, drug treatment and recording of measures related to the management of type 2 diabetes; and to assess whether recommended targets can be met.

**Methods:**

The population comprised subjects registered between 1994 and 2001 with 74 general practices in England and Wales which routinely contribute to the Doctors' Independent Network database. Approximately 500,000 patients and 10,000 type 2 diabetics were registered in each year.

**Results:**

Type 2 diabetes prevalence rose from 17/1000 in 1994 to 25/1000 in 2001. Drug therapy has changed: use of long acting sulphonylureas is falling while that of short acting sulphonylureas, metformin and newer therapies including glitazones is increasing. Electronic recording of HbA1c, blood pressure, cholesterol and weight have risen steadily, and improvements in control of blood pressure and cholesterol levels have occurred. However, glycaemic control has not improved, and obesity has increased. The percentage with a BMI under 25 kg/m^2 ^fell from 27.0% in 1994 to 19.4% in 2001 (p < 0.001).

**Conclusion:**

Prevalence of type 2 diabetes is increasing. Its primary care management has changed in accordance with best evidence. Monitoring has improved, but further improvement is possible. Despite this, glycaemic control has not improved, while the prevalence of obesity in the diabetic population is rising.

## Background

Type 2 diabetes is a common condition with high morbidity and mortality. Its diagnosis appears to be increasing [1-3], probably reflecting an underlying rise in prevalence. In many countries, guidelines for its management have been issued [4-6], and in the United Kingdom (UK), these are reflected in the National Service Framework for Diabetes, which sets national standards and defines service models for the condition[7]. UK Family Practitioners' remuneration will increasingly depend on hitting targets which encompass both process of care and health related outcomes[8]. In this context, process includes monitoring of the disease and of modifiable risk factors that might lead to complications. In particular, it is important to manage cardiovascular risk in diabetics because diabetes accelerates vascular occlusion and much of the excess mortality is due to cardiovascular mortality[9].

Practitioners will be judged largely on the achievement of target levels for the process measures monitored. Achieving such targets requires pharmacotherapy in most cases. In recent years a number of new drugs for the management of diabetes have appeared and research evidence suggests that these should replace some older drugs and supplement others, whilst some drugs, such as metformin, should be used more frequently [10-12].

In this study, we use routinely collected computer data to examine trends in the prevalence of diagnosed Type 2 diabetes, the drug treatment of the condition and recording of measurements utilised in its management, including body mass index (BMI), blood pressure and glycosylated haemoglobin (HbA1c). Finally we examine trends in the achievement of management target levels for these measurements as suggested by national guidance.

## Methods

The Doctors' Independent Network (DIN) is an anonymised, computerised UK primary care database comprising 142 Family Practices that we consider to be good quality data providers[13]. Morbidity and drug data are coded using Read codes (4 byte). This study uses data from 74 practices that had continuous recording from 1994 to 2001, eliminating possible spurious trends caused by having different practices in the sample over time. We included patients in a given year if they remained registered on 31^st ^December of that year and for 6 months previously.

Diabetics were identified by electronically searching the database for all diabetic Read codes. Diabetics on insulin were classified as type I or type II based on an algorithm (available from authors) which used date of diagnosis and date of first being seen in the practice, in combination with the timing of treatment with oral hypoglycaemics (British National Formulary (BNF) section 6.1.2) and/or insulin (BNF section 6.1.1). The decision was largely straightforward for diabetics newly diagnosed while they were registered with practices using their electronic database. Diabetics were classified as type 1 if a Read code "Diabetes + ketoacidosis -no coma" was present or if insulin was given within 90 days of first diagnosis. The problems arose for those diabetics diagnosed in the past who were using insulin at the point of registration with the practice or when the practice started using the electronic database. While dates of first diagnosis were usually available, as were date of registration and first recording of events within practice, we did not have information on when insulin treatment was started prior to electronic recording. In this instance it was necessary to base the decision on the age of the patient at first diagnosis (≥35 years implies type 2) as well as the time lapse between when the patient was first seen in the practice and when an insulin prescription was issued (>180 days implies type 2). Based on a random sample of 150 records of diabetics receiving insulin, the algorithm had 94% sensitivity and 93% specificity for classifying type 2 diabetics compared to blind assessment by a clinician (SDeW) of the same electronic records. Type 2 diabetics were further classified by treatment received in a given year into: diet, oral only, or insulin (with or without oral).

The records of Type 2 diabetics were searched for Read codes for Body Mass Index (BMI), blood pressure, total cholesterol, and glycosylated haemoglobin (HbA1c). Read codes for these data may be indicative (e.g. "cholesterol raised") or have a numerical value assigned. In earlier years indicative codes tended to be used more, and for HbA1c, these were the only recording option before 1997. If an indicative code (or a biologically implausible numerical code) was used then we counted the measurement as having occurred, but set its value to be missing.

Where multiple measurements took place in a given year, we used the last recorded measurement in that year. Numerical values were selected in preference to indicative codes even if they were not the last recorded measurement in a year. BMI values were supplemented by weight records, if an existing height measurement was present in the record, allowing BMI to be calculated.

In order to examine trends in the achievement of management targets, data were compared with targets set in guidance published by the National Institute for Clinical Excellence (NICE). The NICE targets are: BMI <25 kg/m^2^, blood pressure <140/80 mmHg or <160/100 mmHg depending on coronary event risk, HbA1c <6.5% or <7.5% depending on coronary event risk, and total cholesterol <5 mmol/l[4,5]. No target figure is set for smoking reduction.

To test whether any trends in the achievement of management targets were influenced by the improvements in the recording of data, we restricted the analyses to 16 practices with a consistently high level of recording and low levels of missing values. To establish whether any trends in the percentage of diabetics meeting targets were due to differences in the way that newly diagnosed diabetics were being managed, analyses were also repeated after restriction to newly diagnosed diabetics.

### Statistical methods

**Figure 3:**
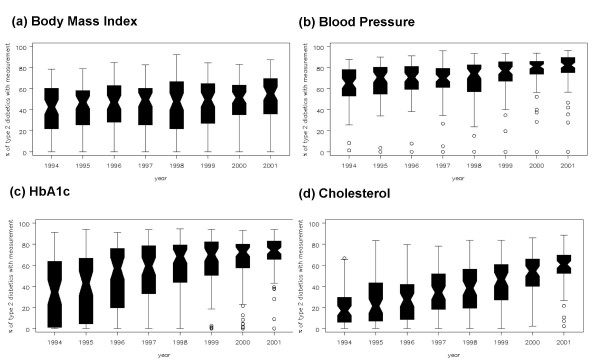
**Distribution of the practice percentage of diabetics with a risk factor measured by year**. – Boxes indicate the median, lower and upper quartiles Whiskers extend to the practice immediately proceeding 1.5 times the interquartile range from the median. Practices lying outside this range are individually plotted. – % of all measurements that were numeric and valid were for each risk factor: BMI (95%), Blood Pressure (97%), HbA1c (61%) and Cholesterol (91%).

To adjust for the changing age structure of type 2 diabetics between 1994–2001 we used the 2001 population of DIN to age standardise prevalence rates using the direct method. Descriptive analyses of practice variation in recording of risk factor data (Figure 3) are summarised using box and whisker plots using the SAS procedure BOXPLOT (SAS Institute Inc., North Carolina, USA). Tests for trend across years are based on fitting the logit of the percentage of diabetics in a practice with the relevant risk factor recorded in a given year using SAS procedure GENMOD.

Tests for trends of targets met across years was based on individual data using a logit link adjusting for age, sex and practice, which was included as a categorical variable with 74 levels. Year was fitted as a linear variable to test for trend. The model was fitted using SAS procedure GENMOD with a first order autoregressive structure to allow for higher correlation of measurements on the same individuals when measured in adjacent years.

In order to assess whether changes in HbA1c over years were influenced by the changes in BMI, we regressed the recorded HbA1c levels on age, sex, practice and year before further including BMI. Various models were fitted using the SAS procedure MIXED, allowing us to take account of individuals contributing data to variable numbers of years.

## Results

### Diabetes prevalence trends

The population totalled approximately half a million patients annually (Table 1). Between 1994 and 2001, the prevalence of diagnosed Type 2 diabetes increased steadily, rising from 18 to 27 per 1000 in men and from 16 to 23 per 1000 in women. The age standardised rates (Table 1) were almost identical. In both sexes, the most notable increases were in the 65–74 age group (men increased from 68 to 101 per 1000, women from 47 to 73 per 1000).

**Table 1:** Prevalence rates (per 1000) of type 2 diabetics from the DIN database (74 practices)

	**1994**	**1995**	**1996**	**1997**	**1998**	**1999**	**2000**	**2001**
*Total males*	*237,872*	*239,427*	*243,618*	*251,005*	*255,833*	*259,670*	*260,383*	*262,290*
- Diet controlled only	1,689	1,788	1,830	1,940	1,971	2,086	2,218	2,285
- On oral drug only	2,111	2,186	2,374	2,573	2,833	3,128	3,417	3,779
- On insulin	542	569	637	704	785	894	985	1,052
**Total type 2 diabetics**	**4,342**	**4,543**	**4,841**	**5,217**	**5,589**	**6,108**	**6,620**	**7,116**
0–34 years	1	1	1	1	1	1	1	1
35–44	7	7	7	8	8	8	9	9
45–54	18	18	19	21	22	23	26	27
55–64	45	47	49	50	52	54	56	57
65–74	69	71	75	79	85	91	96	101
75–84	72	78	77	82	86	94	99	109
85+	79	72	74	72	71	77	81	89
**Crude overall rate**	**18**	**19**	**20**	**21**	**22**	**23**	**25**	**27**
Age Std Rate*	19	20	21	22	23	24	26	27
95% CI	18–21	19–21	20–22	21–23	22–24	23–25	25–27	26–28

*Total females*	*241,995*	*242,996*	*246,885*	*253,383*	*258,695*	*262,241*	*263,475*	*264,763*
- Diet controlled only	1,404	1,496	1,565	1,658	1,722	1,791	1,885	2,065
- On oral drug only	1,824	1,915	2,070	2,244	2,427	2,586	2,844	3,048
- On insulin	528	532	579	632	712	801	888	941
**Total type 2 diabetics**	**3,756**	**3,943**	**4,214**	**4,534**	**4,861**	**5,178**	**5,617**	**6,057**
0–34 years	1	1	1	1	1	2	2	2
35–44	6	6	6	7	7	8	9	9
45–54	12	13	14	15	15	16	17	19
55–64	33	34	34	34	36	38	38	40
65–74	47	50	53	59	63	66	70	73
75–84	53	56	59	59	61	67	72	76
85+	48	48	53	52	56	58	61	68
**Crude overall rate**	**16**	**16**	**17**	**18**	**19**	**20**	**21**	**23**
Age Std Rate*	16	17	17	18	19	20	22	23
95% CI	15–17	16–18	16–19	17–19	18–20	19–21	21–23	22–24

* - Age standardised rate is made to the 2001 DIN Population

### Trends in therapy

**Figure 1:**
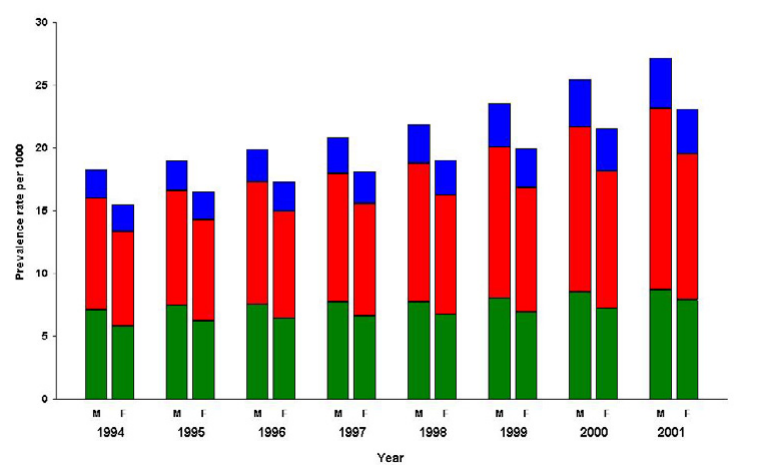
**Prevalence of type 2 diabetic treatment groups in the DIN database over time**. Blue – On insulin, Red – On oral drug only, Green – Diet controlled only

During the 1990's there were steady increases in the prevalence of diet only, oral treatment only and insulin treated type 2 diabetes (Figure 1). However, the proportion of type 2 diabetics treated by diet alone fell between 1994 and 2001 (from 38.2% to 33.0%), while those treated with any insulin (13.2% to 15.1%) and oral agents only (48.6% to 51.8%) both increased.

**Figure 2:**
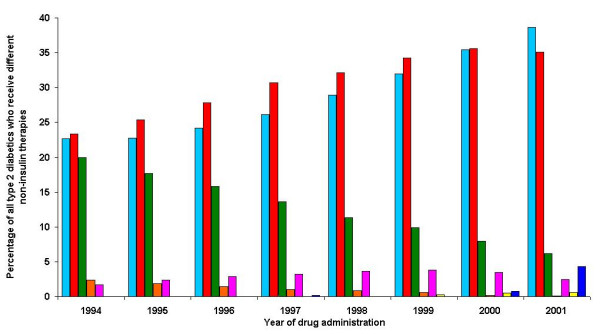
**Changes in the non-insulin treatment of type 2 diabetics over time**. Light Blue – Metformin, Red – Short acting sulphonylurea, Green – Long acting acting sulphonylurea, Orange – Ultra long acting sulphonylurea, Purple – Acarbose, Yellow – Meglitinides, Dark Blue – Thiazolidinediones/Glitazones **Footnote: **Treatment groups are not mutually exclusive

Trends in oral therapy are shown in Figure 2. Use of ultra-long acting sulphonylureas had almost completely ceased by 2001 when it was received by 0.1% of diabetics. The use of long acting sulphonylureas and guar gum (0.2% in 1994 to 0.04% in 2001 – not shown in Figure 2) has also reduced. In contrast, the use of short acting sulphonylureas increased (from 23.3% to 35.1%), as did the use of metformin (from 22.6% to 38.9%). The use of the intestinal alpha glucosidase inhibitor, acarbose, increased up to 1999, rising from 1.7% in 1994 to 3.8% in 1999, then declined back to 2.4% in 2001. Thiazolidinediones and meglitinides started being used in the final years of the study.

### Recording of indicators of process of care

Trends in recording of measurements linked to the process of diabetic care where targets are set in NICE guidance are shown in Figure 3. Recording of all measures has increased (P < 0.001), especially the recording of HbA1c (practice median 34 % in 1994 to 74% in 2001) and cholesterol (practice median 17% in 1994, 61% in 2001). The practice median for recording of BMI was 43% of patients in 1994 rising to 55% in 2001; for blood pressure it rose from 65% in 1994 to 82% in 2001. Less than 10% of all measurements made were classified as "missing" for all except HbA1c. For HbA1c 39% of measurements recorded as being made were "missing" due to the sole existence of indicative codes before 1997. There was variation in recording between practices in any given year which, while it diminished over time, was still marked in 2001, with one practice still not recording any of the measures.

### Outcomes: achievement of national quality targets

**Figure 4:**
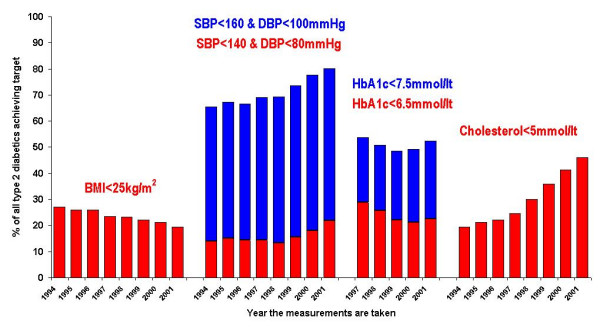
**(NICE/NSF) Risk factor targets achieved in all type 2 diabetics by year**. (Results are age & sex standardised to the 2001 population) Denominators are the number of subjects with a valid numerical value recorded. HbA1C results start in 1997 as numerical codes could not be recorded prior to 1997.

Trends in the percentage of diabetics whose management achieved NICE targets are shown in Figure 4. There has been a marked increase (P < 0.001) in the percentage of diabetics with a total cholesterol <5 mmol/l. (46.2% in 2001), and a steady increase in patients with blood pressure readings below the target levels (P < 0.001), although only 22.5% had a blood pressure less than 140/80 in 2001.

The percentage of patients meeting either of the target HbA1c figures has however tended to fall, except for 2001 when there was a small increase (trend tests P < 0.001 for lower target, P = 0.14 for higher target) : 22.5% of patients in 2001 had an HbA1c of less than 6.5% compared to 28.9% in 1997; 52.3% of patients in 2001 had an HbA1c less than 7.5% compared to 53.7% in 1997. A steady fall in the percentage of patients with a BMI less than 25 kg/m^2 ^has also occurred (P < 0.001) with 19.4% of patients achieving this criterion in 2001, compared with 27.0% in 1994.

In order to assess whether the lack of improvement in glucose control over time might be due to the steady increase in the obesity of the diabetic population, we estimated the mean HbA1c levels adjusted for age, sex and practice and then further adjusted for BMI. The mean levels unadjusted for BMI reflected the same pattern as in figure 4; that is the mean HbA1c rose from 7.73% in 1997 to 7.82% in 2000 before falling back to 7.69% in 2001. While BMI was highly significantly related to HbA1c, adjustment for it had little effect on the estimated HbA1c means for the different years.

### Trends in practices with high levels of recording of target related data

When the above analyses were restricted to 16 practices with consistently high levels of data recording (corresponding to those above the upper quartile in Figure 3 for each factor), the trends in meeting targets as described above remained almost unaltered (data not shown).

### Treatment of newly diagnosed diabetics

The trends in treatment and other management in newly diagnosed diabetics were almost identical to those seen for all diabetics (data not shown).

## Discussion

### Principal findings

This study confirms that the prevalence of all diagnosed type 2 diabetes continues to rise. The data are consistent with data published from the General Practice Research Database (GPRD) to 1998[3]. However, that study failed to identify diet treated diabetics or to include those type 2 diabetics treated with insulin. Our figures for overall prevalence and type of therapy amongst type 2 diabetics compares well with the only other UK source we have identified. In that much smaller study, the prevalence of type 2 diabetes was 20.3 per 1000 in males and 16.7 per 1000 in females in 1997, of whom 34% were treated by diet only, 53% were oral only and 14% were on insulin[14]. It is reassuring that two studies based on very different methodologies give such similar results.

The prevalence of diabetes has risen steadily in developed and developing countries throughout the second half of the 20^th ^century[1]. Rising levels of obesity in the general population are believed to be one of the principal drivers[1], and in a recent Danish study it was concluded that the rise in diabetes between 1974/75 and 1996/97 was entirely attributable to the concurrent rise in body mass index[15]. It seems likely that this underlies the steady increase we have observed, given that the percentage of adult males in England with a BMI over 30 rose from 13.8% in 1994 to 21.0% in 2001 while for women the trend was from 17.3% to 23.5% [16]. While there is a theoretical possibility that changes in the definition of diabtetes introduced from 1998 on may have had some effect, it seems likely to have been limited given that the steady increase in the prevalence of type 2 diabetes predated the change in diagnostic criteria; in 1998–9 the definition of diabetes shifted from those with a 2 hour post load plasma glucose > 11.1 mmol/l or a fasting level ≥ 7.8 mmo//l to a fasting plasma glucose ≥ 7 mmol/l[17].

Family doctors have altered drug therapies in accordance with research evidence and best guidance [10-12] and have introduced new drugs as they become available. The broad trends in therapy are consistent with published data from the Prescription Pricing Authority[18], the advantage being that our data are patient rather than prescription based and also specific to type 2 diabetes.

Measurement and electronic recording of data of value in the management of diabetes has steadily increased. The only measurement not to show substantial improvement and an increase to a high level of recording is the BMI. It is not clear why this should be, but improvement could be easily achieved. The steady improvement in data recording predates the issue of national guidance and targets and implies that doctors have improved care through the assimilation of best evidence without the need for other inducements.

It has recently been suggested that quality of data recording is not a valid indicator of quality of care[19] and, notwithstanding improvements in monitoring and in drug therapy, it is striking to note that glycaemic control has not improved over the period of observation. This is paralleled by a decrease in the percentage of patients achieving a BMI of <25 kg/m^2^. The increasing weight of the diabetic population reflects the general increase in weight of the UK population[20], but also may reflect the tendency for many diabetic medications to produce weight gain as an unwanted effect. Data from the US suggest that obesity is rising in newly diagnosed diabetics and that this is associated with poorer prognosis[21]. It is ironic that BMI is the least well recorded measure in this study.

It is tempting to conclude that the lack of improvement in HbA1c control is attributable to the increasing obesity of the diabetic population. However, our analyses of this suggest that the increase in BMI amongst diabetics only had a small effect on the observed HbA1c levels. Further investigation of this issue is certainly warranted, but the statistical analysis is complicated due to the unbalanced data whereby individuals contribute varying numbers of observations, while data recording standards are changing over time, raising the possibility that missing observations are not random. It would also be important to distinguish between changes in BMI as a result of diabetic therapies, and the increasing BMI of the general population.

Other targets in diabetic care are increasingly being met, especially in the area of lipid control. Increased use of drug therapy probably accounts for this and mirrors the increased use of lipid lowering therapy in secondary prevention of ischaemic heart disease[22]. Blood pressure control has improved but is a long way from meeting the lower targets for most patients as suggested by guidance[4]. This may again be consequent on the increasing obesity of the diabetic population and suggests that blood pressure targets in general practice populations may be very difficult to achieve. It should also be noted that blood pressure target levels in UK guidance are rather modest compared to those in US guidance[6].

In contrast to our findings, a recent Swedish study, based on a national diabetes register, reported improvements in HbA1c, as well as in blood pressure levels and increased use of statins, between 1996 and 1999; the Swedish study also reported rising BMI levels [23]. A Dutch study [24] reported similar improvements in blood pressure and cholesterol levels between 1993 and 1999. However, that study also showed improvements in HbA1c. No data on obesity were presented.

Finally, when assessing targets it is important to recognise that complete success is unrealistic. Some patients who comply will not respond to treatment, others will opt-out of treatment, while for others with major co-morbidities the GP may choose not to treat [24]. Further work to assess the realistic target achievable would be worthwhile.

### Limitations of the study

Our study only reflects what was recorded on the computer systems. It seems reasonable to focus on such data given that these are increasingly being used for monitoring performance in achieving targets. However, it is important to consider whether a rising level of recording or the changing population of diabetics might account for the changes observed. As similar trends were seen in a subset of practices that had always had high levels of recording and low levels of missing values, it is unlikely that rising levels of recording explain our observations. This is consistent with the findings regarding data completeness and quality of care previously alluded to[19]. Comparable trends were also seen in new as well as established diabetics and observed in the control of blood pressure and cholesterol in patients with cardiovascular disease; suggesting that there is consistent improvement in the control of these risk factors.

With DIN, as with all primary care databases, it is important to consider whether the prevalence of disease, recording of risk factors and achievement of targets are nationally representative. The age-sex structure of DIN is identical to the UK average, but more practices are in the south and lower socio-economic groups are under-represented[13]. Nevertheless we have demonstrated that period prevalence rates for a wide range of diseases are similar to those in GPRD[22,25], and this also holds for diabetes (data not shown). There is also evidence that research and non-research practices are similar in disease outcomes[26]. It seems likely that levels of electronic risk factor recording will be above average in an electronic database, although many practices were still doing poorly. While in theory higher levels of recording might improve control of risk factors, there was no clear evidence of this within DIN practices – at practice level there was no association between level of recording and the lower management target for HbA1c (Pearson correlation = 0.07 in 2001). In summary we believe that the trends seen are typical of those seen nationally, though the overall level of electronic recording is likely to be higher in DIN.

## Conclusion

This study highlights the improvements in the process of diabetic care that have been achieved in primary care without the inducements of national targets. Despite this, glycaemic control has not improved and obesity has increased. While increasing obesity does not straightforwardly explain why glycaemic control is not improving it is of itself associated with worse prognosis[21]. Doctors have limited power to affect the weight of their patients[20], and unless weight control in the general population becomes a matter of more importance in national policy, it seems likely that the incidence of diabetes will continue to rise while targets for diabetic control will not be met in most patients.

## Competing interests

SdeL is a member of the DIN board – DIN is a registered charity. NR is a director of a company that provides DIN data commercially.

## Authors' contributions

SdeL and DC conceived the idea for this paper. CS and IC were responsible for the analyses under the direction of DC. NR advised on issues relating to the DIN database. SdeL, SDeW and DC wrote the paper. All authors commented on drafts of the paper

## Pre-publication history

The pre-publication history for this paper can be accessed here:


